# Electroporation and genetic supply of Cas9 increase the generation efficiency of CRISPR/Cas9 knock-in alleles in C57BL/6J mouse zygotes

**DOI:** 10.1038/s41598-020-74960-7

**Published:** 2020-10-21

**Authors:** Samy Alghadban, Amine Bouchareb, Robert Hinch, Polinka Hernandez-Pliego, Daniel Biggs, Chris Preece, Benjamin Davies

**Affiliations:** 1 Wellcome Centre for Human Genetics, University of Oxford, Roosevelt Drive, Oxford, OX3 7BN UK; 2 Big Data Institute, Li Ka Shing Centre for Health Information and Discovery, University of Oxford, Oxford, OX3 7LF UK

**Keywords:** Genetic engineering, Model vertebrates, Mouse, Animal biotechnology, Genetic engineering

## Abstract

CRISPR/Cas9 machinery delivered as ribonucleoprotein (RNP) to the zygote has become a standard tool for the development of genetically modified mouse models. In recent years, a number of reports have demonstrated the effective delivery of CRISPR/Cas9 machinery via zygote electroporation as an alternative to the conventional delivery method of microinjection. In this study, we have performed side-by-side comparisons of the two RNP delivery methods across multiple gene loci and conclude that electroporation compares very favourably with conventional pronuclear microinjection, and report an improvement in mutagenesis efficiency when delivering CRISPR via electroporation for the generation of simple knock-in alleles using single-stranded oligodeoxynucleotide (ssODN) repair templates. In addition, we show that the efficiency of knock-in mutagenesis can be further increased by electroporation of embryos derived from Cas9-expressing donor females. The maternal supply of Cas9 to the zygote avoids the necessity to deliver the relatively large Cas9 protein, and high efficiency generation of both indel and knock-in allele can be achieved by electroporation of small single-guide RNAs and ssODN repair templates alone. Furthermore, electroporation, compared to microinjection, results in a higher rate of embryo survival and development. The method thus has the potential to reduce the number of animals used in the production of genetically modified mouse models.

## Introduction

CRISPR/Cas9 site-specific nucleases have greatly facilitated the production of genetically modified mouse models harbouring specific mutations^1^. The Cas9 nuclease can be programmed via a small single guide-RNA (sgRNA) and, when introduced into the fertilized zygote, generates a double-strand break at the site-of-interest, defined by sequence homology at the 5′ of the sgRNA. Aberrant repair of this break results in the introduction of insertion or deletion (indel) mutations which can be sufficient to disrupt a gene’s function, generating knock-out alleles. Alternatively, through the co-delivery of a repair template, directed change to the genomic sequence at the target gene can be achieved, generating knock-in alleles. Initial reports delivered the CRISPR/Cas9 machinery by microinjection and efficient generation of knock-out, knock-in and floxed mouse models was reported^2,3^.

As an alternative to delivery by microinjection, electroporation can be used as an efficient delivery method for CRISPR/Cas9 reagents. This was first demonstrated using rat embryos^4^ and was subsequently shown also to be an effective delivery method for mouse embryos^5,6^. The first studies delivered Cas9 as mRNA^4–6^, but more recent reports have shown that delivery of CRISPR/Cas9 prepared as ribonucleoprotein (RNP) can be achieved by electroporation^7–12^. The delivery of the reagents as RNP leads to more immediate activity which has been shown to increase mutagenesis efficiencies^9^ and decrease the level of mosaicism seen within the engineered zygote^8^.

An initial direct comparison of pronuclear microinjection with electroporation for the production of indel mutations at a number of loci, concluded that pronuclear microinjection was a more reliable method of model production^5^. However, the authors themselves admit that the comparison was flawed as the pronuclear microinjection experiments were performed on inbred NOD/ShiLtJ embryos whereas the electroporation experiments were performed on hybrid B6D2F2/J zygotes, and are thus not directly comparable. A more recent study performed a more systematic comparison between delivery methods in inbred C57BL/6N embryos and concluded electroporation was considerably more efficient, and observed an average three-fold increase in the production efficiency of exonic deletion alleles^13^.

In this study we perform a systematic side-by-side comparison of electroporation versus microinjection in C57BL/6J embryos and extend the comparison for the production of both indel and small knock-in alleles. We demonstrate that efficiencies of mutagenesis following electroporation are broadly similar to those obtained using pronuclear microinjection as the delivery mechanism, but report a significant efficiency improvement for electroporation when generating simple knock-in alleles, using data collected at multiple independent genomic loci.

We and others have shown previously that transgenic Cas9-expressing embryo donors can be used effectively for mouse model production, obviating the need to provide exogenous Cas9^14–16^. Maternally contributed Cas9 protein within the fertilized zygote is sufficient for mutagenesis, and only the sgRNA and repair template need be introduced. Indeed, maternally supplied Cas9 was found to result in a more consistent and efficient mutagenesis^14,16^. In this study we extend our side-by-side comparison of delivery methods to examine the effect of introducing sgRNA and repair template into zygotes derived from Cas9-expressing females by electroporation. Using this approach, which involves only the delivery of small nucleic acids, we report a further increase in the production efficiency of small knock-in alleles.

## Results

### Establishing the parameters for side-by-side comparisons of electroporation versus pronuclear microinjection

**Figure 1:**
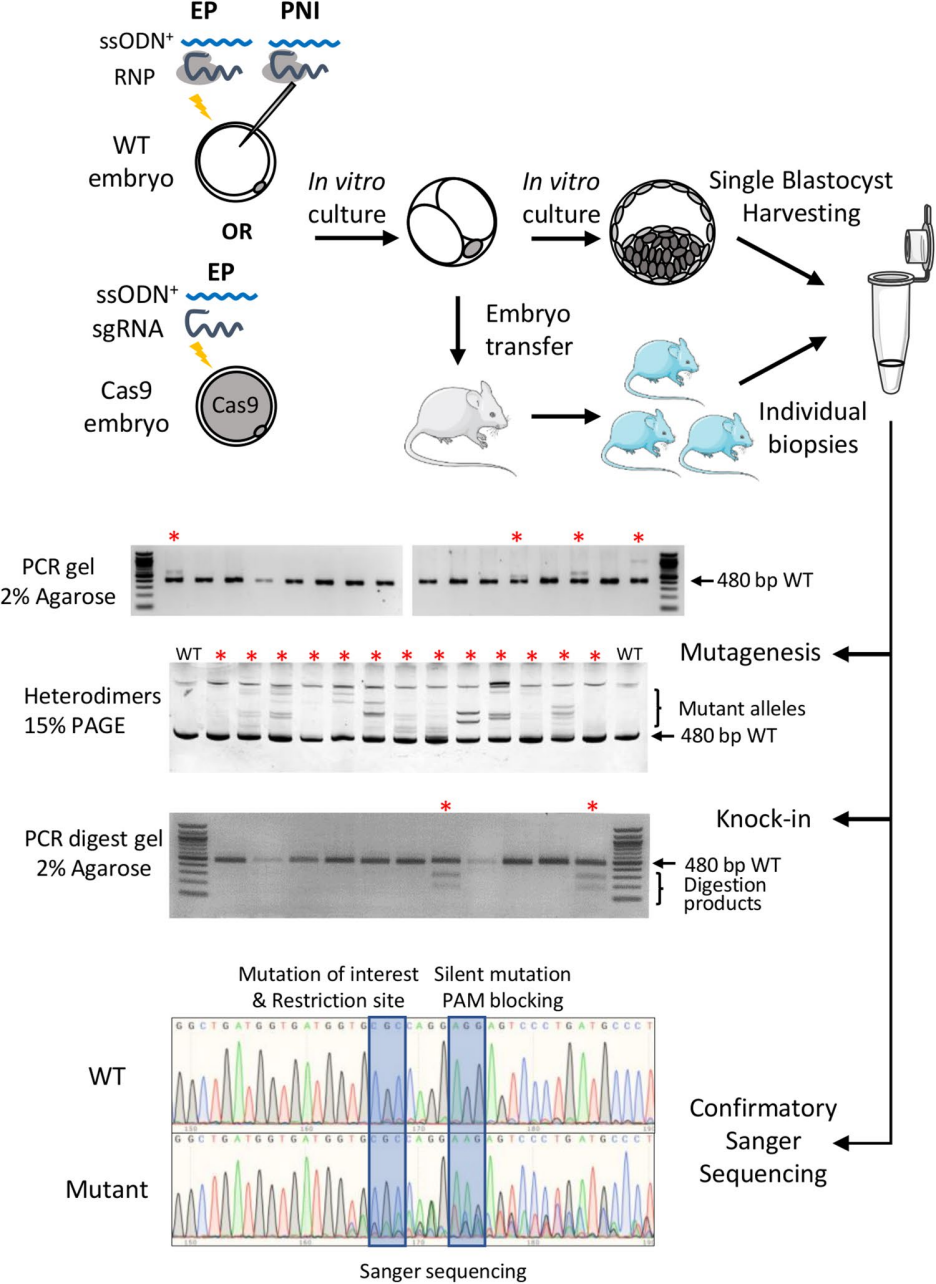
Experimental design of the three-way comparison study. Schematic experimental design to assess the total mutagenesis and knock-in rate induced in embryos or live pups obtained from wild-type C57BL/6J zygotes or maternally supplied Cas9 C57BL/6J zygotes after microinjection or electroporation of CRISPR reagents. ssODN^+^: optional inclusion of ssODN in the electroporation mix.

**Figure 2:**
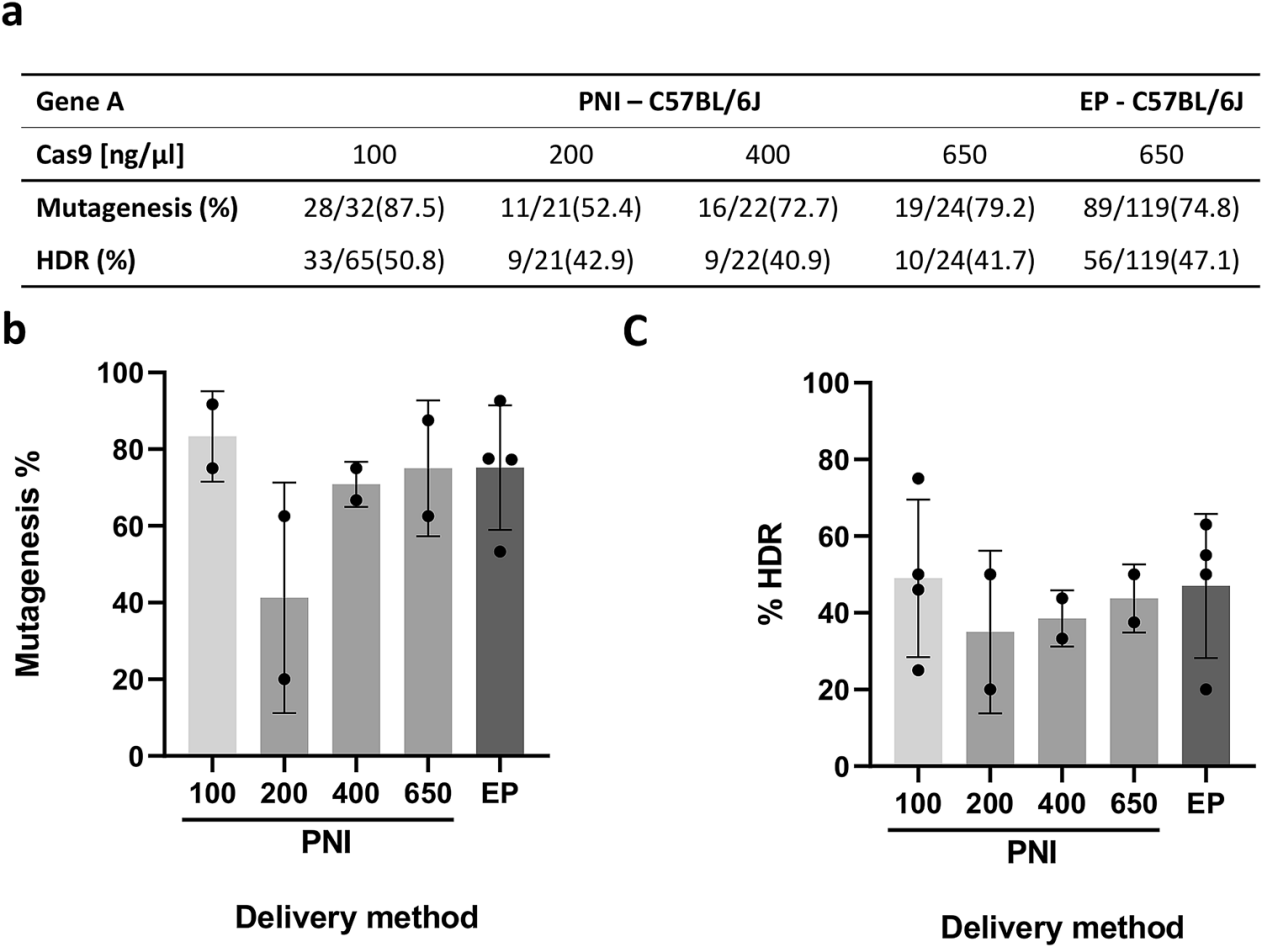
Proof-of-principle and conditions optimization experiments. (**a**) Table summarizing editing of Gene A after pronuclear microinjection (PNI) or electroporation (EP) of CRISPR reagents into wild-type C57BL/6J embryos with various concentrations of Cas9 protein. **(b)** Total mutagenesis rate at Gene A locus. **(c)** Rate of homology directed repair at Gene A locus. The values presented in table (**a**) are total numbers across all replicates, whereas the numbers from individual replicate experiments are shown in (**b**) and (**c**).

We first designed a proof-of-principle experiment to confirm that our experimental settings were suitable for efficient mutagenesis of mouse zygotes by electroporation using previously reported optimized conditions^10^ (130 ng/µl sgRNA, 650 ng/ul recombinant NLS-Cas9 protein, 430 ng/µl ssODN). Electroporated C57BL6/J embryos were cultured to the blastocyst stage then lysed and genotyped for the presence of indel mutations, by heteroduplex analysis of PCR amplicons with non-denaturing polyacrylamide gel electrophoresis (PAGE), or for successful knock-in mutation, by restriction endonuclease digestion of the PCR amplicons, signalling the incorporation of a diagnostic restriction site, followed by confirmatory Sanger sequencing (Fig. 1). An average mutagenesis rate of 74.8% and a knock-in efficiency of 47.1% were observed, confirming the conditions were suitable for efficient production of both indel and knock-in alleles (Fig. 2a).

The aim of this study was to compare electroporation as a delivery method side-by-side with conventional pronuclear microinjection. To allow meaningful comparisons, it was first necessary to establish the optimal concentration of CRISPR/Cas9 reagents delivered by pronuclear microinjection. To this end, we microinjected C57BL6/J embryos with CRISPR/Cas9 reagents, using a range of concentrations of recombinant NLS-Cas9 protein, and established that a concentration of 100 ng/μl was optimal for both overall mutagenesis and knock-in rates (Fig. 2b,c). Furthermore, this concentration yielded the highest rate of development from 2-cell embryos to the blastocyst stage (Supplementary Table 1). We selected 100 ng/μl as the optimal concentration which is in good agreement with our previous work^14^ and values reported in the literature^17,18^. Efficiencies of mutagenesis for both indel and simple knock-in alleles via electroporation were very similar to those obtained via pronuclear microinjection, using the optimal selected concentration.

### CRISPR delivery via electroporation shows improved efficiencies for the production of knock-in alleles

**Figure 3:**
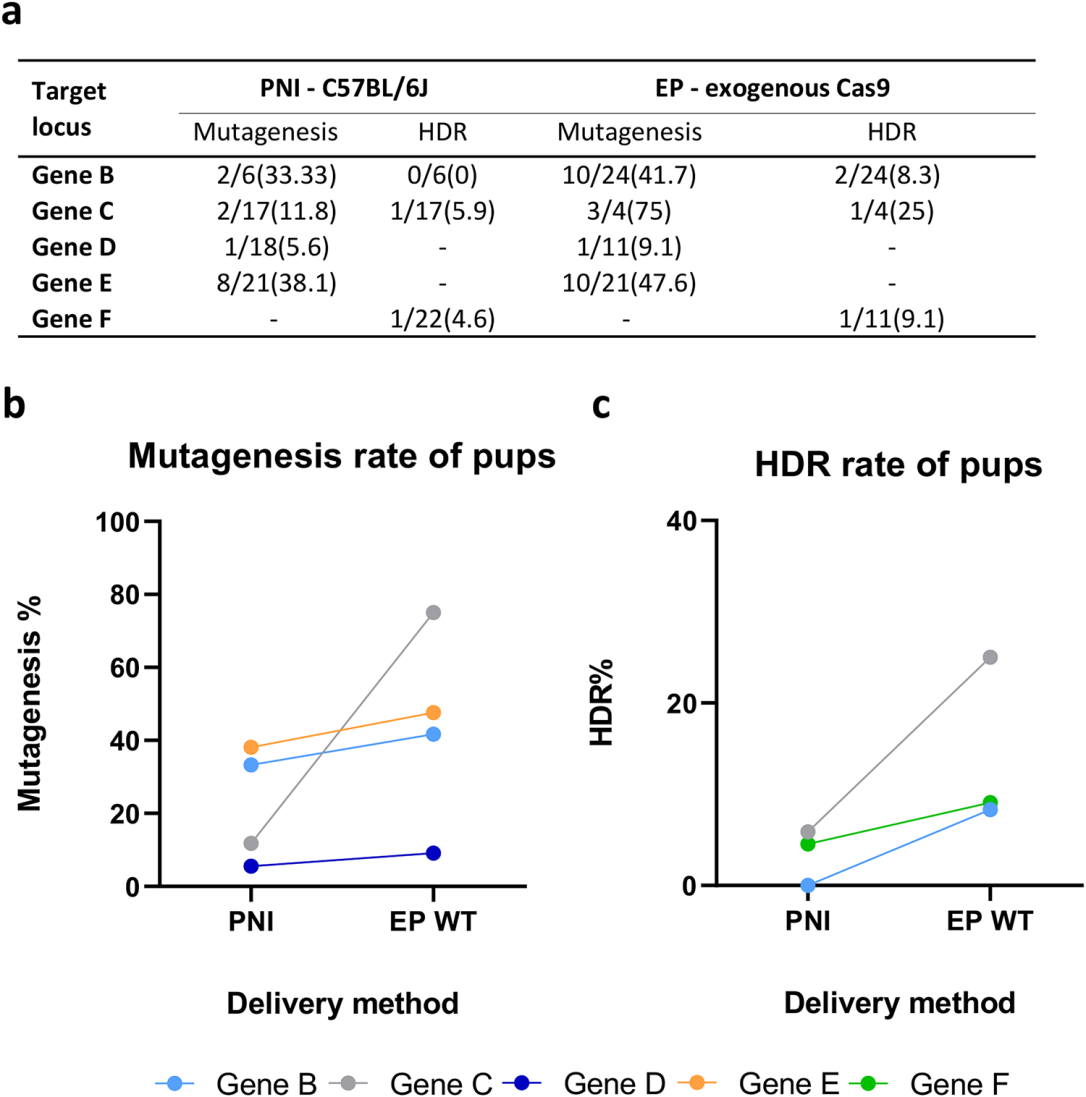
Comparison of pronuclear microinjection versus electroporation (live pups). (**a**) Table summarizing editing efficiency at 5 gene loci in live pups after pronuclear microinjection (PNI) or electroporation (EP) of CRISPR reagents into wild-type C57BL/6J zygotes. (**b**) Total mutagenesis rate at 4 gene loci in live pups obtained from pronuclear microinjection (PNI) or electroporation (EP WT) of CRISPR reagents into wild-type C57BL/6J zygotes. (**c**) Rate of homology directed repair at 3 gene loci. Colours representing individual target loci are consistent across the figures (Supplementary Table 4).

Having demonstrated that electroporation could be as effective as pronuclear microinjection as a delivery method for CRISPR reagents in vitro, we asked the question whether the method could be applied robustly across multiple genomic loci for the production of genetically modified pups. RNP preparations of CRISPR/Cas9 targeting five different gene loci (Supplementary Table 2) were each electroporated and microinjected (pronuclear) into groups of fertilized C57BL/6J oocytes to allow a side-by-side comparison of the two delivery methods. Where appropriate, ssODN repair templates (Supplementary Table 3) were included in the microinjection/electroporation mix. Treated embryos were cultured overnight to the 2-cell stage and then transferred to pseudopregnant recipient females (Supplementary table 4). Live offspring were genotyped for the presence of indel mutations as described above (Fig. 3a).

For all of the targeted genes where total mutagenesis was assessed (gene loci n = 4) the rate observed in the resulting pups was increased by electroporation delivery, when compared with pronuclear microinjection (Fig. 3b), although on a gene-by-gene basis this improvement was not statistically significant (Fisher test, *p* > 0.05 for each gene locus; see also Supplementary Table 6). For experiments where an ssODN repair template was included (gene loci n = 3), electroporation also led to an increase in knock-in efficiency compared with microinjection (Fig. 3c) across all three target loci, but again the improvement observed for each gene failed to reach statistical significance (Fisher test, *p* > 0.05 for each gene locus; see also Supplementary Table 6).

For a complete comparison of the two delivery methods, we analysed the data taking into account all tested loci using a mixed-effect logistic regression model, which uses the two methods (pronuclear injection vs. electroporation) and the experimental assessment (genotyping blastocysts or live pups) as fixed effects and the gene loci as random-effects. Using this model, electroporation showed a trend towards increased efficiency in overall mutagenesis rate when compared with microinjection, however, this failed to reach statistical significance (β = 0.1448; *p* value = 0.662). However, when considering the generation of knock-in alleles, a statistically significant improvement of 87% in the average rate of knock-in allele production for electroporation versus microinjection was observed (β = 0.6282; *p* value = 0.0465).

In summary, analysing all observations across a number of different genomic loci, our data demonstrates that electroporation and pronuclear microinjection are very comparable with respect to overall mutagenesis efficiency. Furthermore, the data suggests that electroporation leads to a significant improvement in the generation efficiency of knock-in alleles when compared with conventional pronuclear microinjection (see summary of all data in Fig. 6).

### Efficient indel and knock-in allele production via electroporation of sgRNA and ssODN components in combination with maternally supplied Cas9

We previously reported that maternal contribution of Cas9 to the zygote (through the use of donor female mice which ubiquitously express Cas9), facilitates mutagenesis when using classical pronuclear microinjection^14^. We were thus interested to explore the consequences of using such zygotes preloaded with maternal Cas9 for mutagenesis experiments using electroporation as a delivery method. We hypothesized that a potential advantage is that only the nucleic acid components of the CRISPR system, i.e. the sgRNA and, if required, the ssODN repair template, need be delivered to the zygotes and this could potentially impact mutagenesis efficiencies.

To explore the feasibility and any potential efficiency improvements when using electroporation in combination with genetic Cas9 supply, we electroporated sgRNA and ssODN repair templates into zygotes prepared from heterozygous Cas9-expressing female mice, and compared the outcome with electroporation of RNP and ssODN repair templates into wild-type C57BL/6J zygotes. For an initial assessment, the efficiency of the production of site-specific mutant alleles was investigated at six different gene loci (Supplementary Table 5) and the resulting zygotes were cultured in vitro to the blastocyst stage, lysed and genotyped.

**Figure 4:**
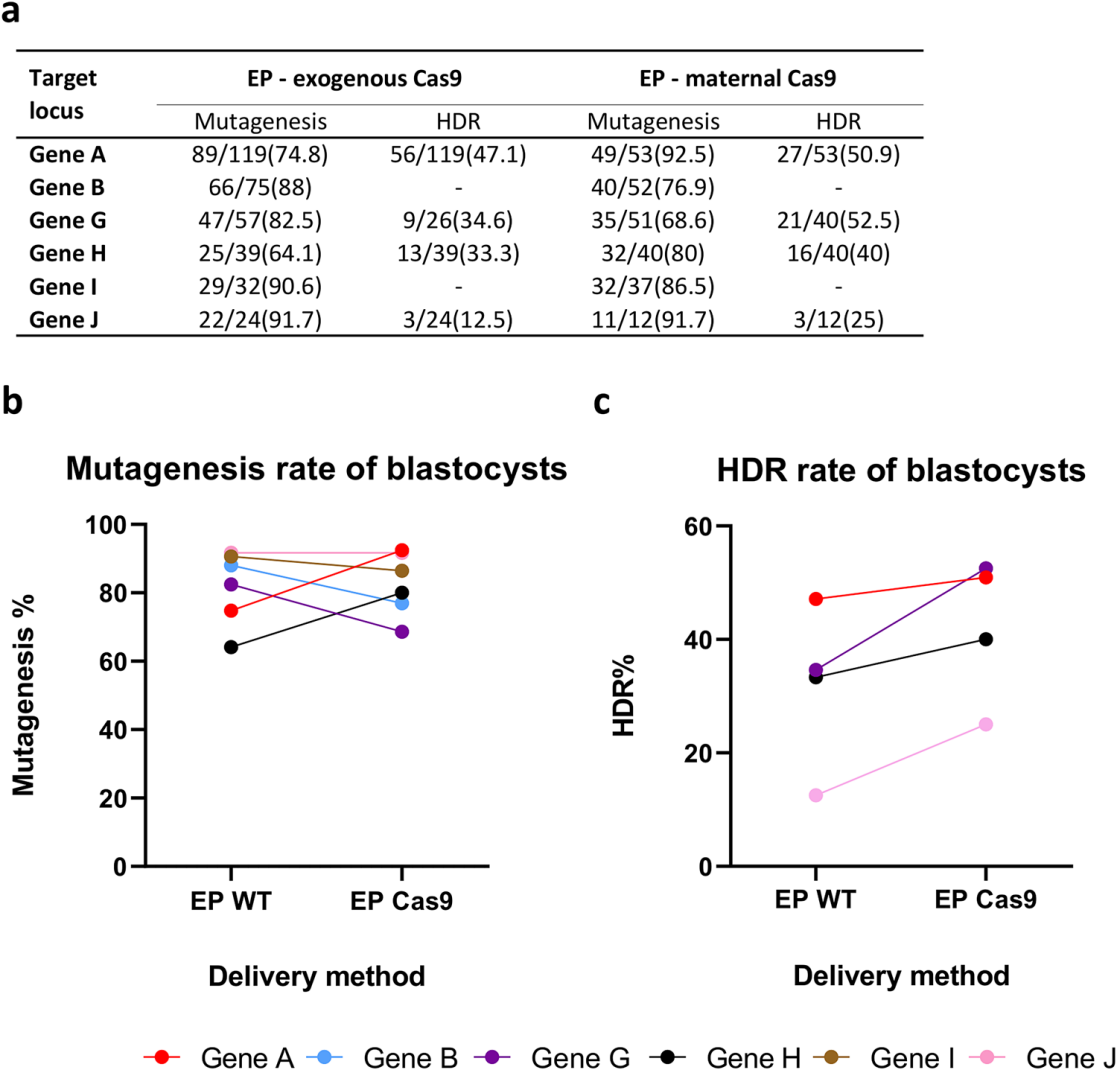
Comparison of electroporation with exogenously or maternally supplied Cas9 in blastocyst. (**a**) Table summarizing editing efficiency at target sites for 6 gene loci in embryos cultured to the blastocyst stage after electroporation (EP) of CRISPR reagents into zygotes harvested from wild-type C57BL/6J females (exogenous Cas9) or Cas9-expressing females (maternal Cas9). (**b**) Total mutagenesis rate at 6 gene loci for electroporation delivery using either exogenous (EP WT) or maternally supplied Cas9 (EP Cas9). (**c**) Rate of homology directed repair at 4 gene loci. Colours representing individual target loci are consistent across the figures (Supplementary Table 5).

These initial experiments confirmed that Cas9 supplied by the mother was sufficient for efficient production of both indel and knock-in alleles when delivering the sgRNA and ssODN repair templates by electroporation (Fig. 4a). Indeed, comparable rates of mutagenesis were seen whether delivering exogenous Cas9 to wild-type zygotes, or whether relying on maternally contributed Cas9 (Fig. 4b), and on a gene-by-gene basis no statistically significant differences were found (Fisher test, *p* > 0.05 for each gene locus; see also Supplementary Table 6). Interestingly, for experiments where an ssODN repair template was introduced (gene loci n = 4), the generation efficiency of knock-in alleles was increased with maternally contributed Cas9 outperforming exogenous delivery of Cas9 (Fig. 4c) at all loci tested. Although individual comparisons at the level of individual gene loci failed to reach statistical significance (Fisher test, *p* > 0.05 for each gene locus), a marginally significant improvement in overall knock-in efficiency was seen when considering all the gene loci together (Chi squared, *p* = 0.046, Supplementary Table 6), although the large variability in efficiency caused by gene locus complicates this analysis approach (see below for a more rigorous approach).

### Electroporation of zygotes with maternally supplied Cas9 leads to improvements in knock-in efficiency

**Figure 5:**
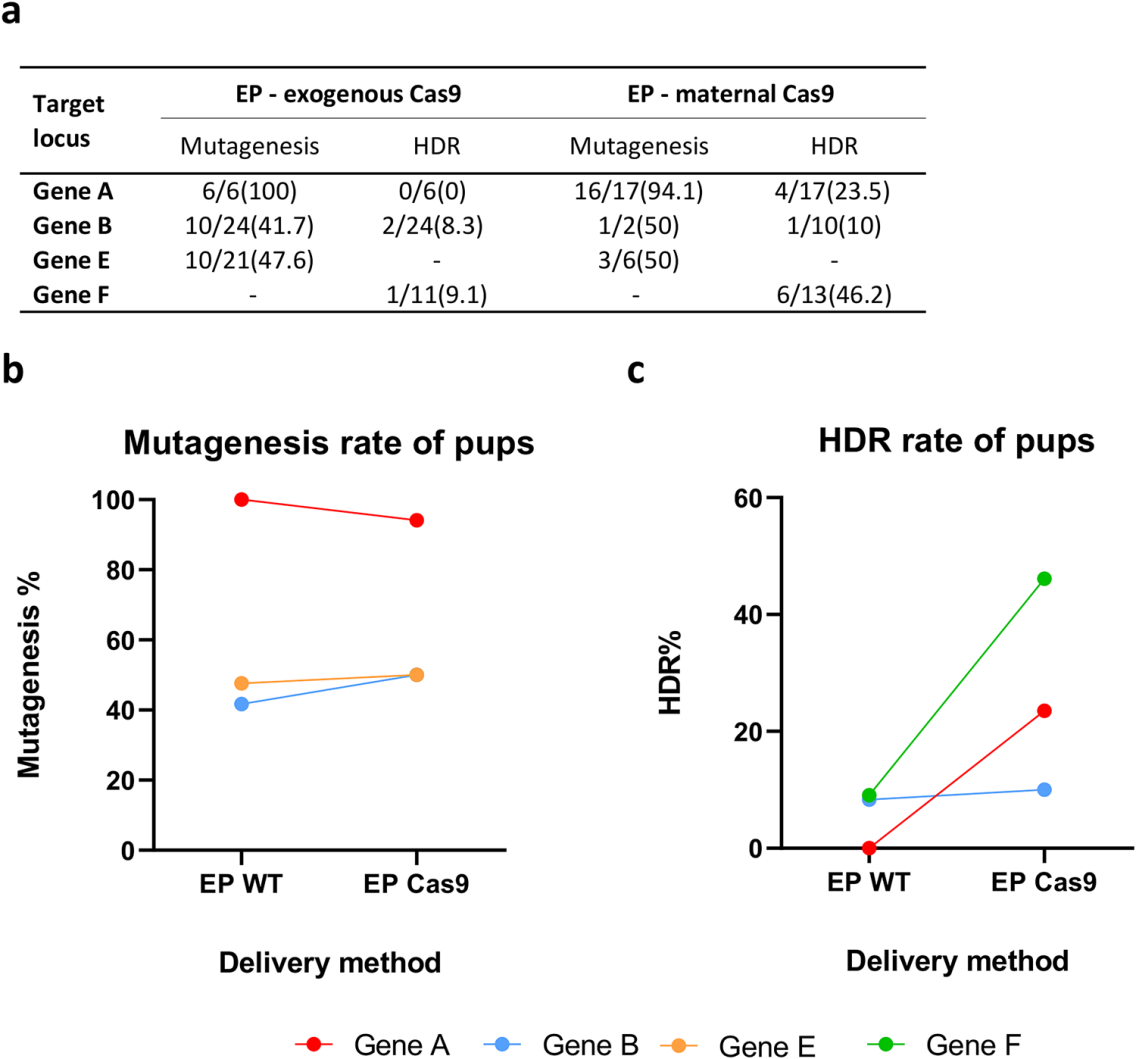
Comparison of electroporation with exogenously or maternally supplied Cas9 in live pups. (**a**) Table summarizing editing efficiency at 4 gene loci in live pups after electroporation (EP) of CRISPR reagents into embryos harvested from wild-type C57BL/6J females (exogenous Cas9) or Cas9-expressing females (maternal Cas9). (**b**) Total mutagenesis rate at 3 gene loci for electroporation delivery with either exogenous (EP WT) or maternally supplied Cas9 (WP Cas9). (**c**) Efficiency of homology directed repair at 3 gene loci. Colours representing individual target loci are consistent across the figures (Supplementary Table 4).

To verify this preliminary result from in vitro cultured embryos, we performed further side-by-side comparisons of sgRNA/ssODN electroporation into zygotes prepared from Cas9-expressing female mice, or RNP/ssODN electroporation into C57BL/6J zygotes, addressing a number of different gene loci, and transferred the embryos to foster mothers and allowed them to develop to term (Supplementary Table 4). A very similar picture emerged in the genotypes of the resulting pups to the results of our in vitro cultured embryos (Fig. 5a), with overall mutagenesis rate being essentially unchanged when comparing exogenous versus maternal Cas9 supply (Fig. 5b). However, relying upon maternal Cas9 supply led to an increase in the frequency of knock-in mutations (Fig. 5c). Although these increases were not found to be statistically significant on a gene-by-gene basis (Fisher test, *p* > 0.05, for each gene locus), a significant improvement in overall efficiency was seen when considering all the gene loci together (Chi squared, *p* = 0.0163), although again the large variability in efficiency caused by gene locus complicates this analysis approach.

For a more rigorous exploration of whether efficiency improvements were apparent, we applied a similar mixed-effect logistic regression model as previously, which takes into consideration all the observations across all the genes tested, using the two methods (electroporation of exogenous RNP /ssODN into wild-type C57BL/6J zygotes or the electroporation of sgRNA/ssODN into maternally supplied Cas9 zygotes) and the experimental assessment (genotyping blastocysts or live pups) as fixed effects and the gene loci as random-effects. Using this model, the mutagenesis rate seen using either maternally supplied or exogenous delivered Cas9 following electroporation was unchanged (β = 0.2787 *p* value = 0.1657), as expected from the pattern of data (Figs. 4b and 5b).

**Figure 6:**
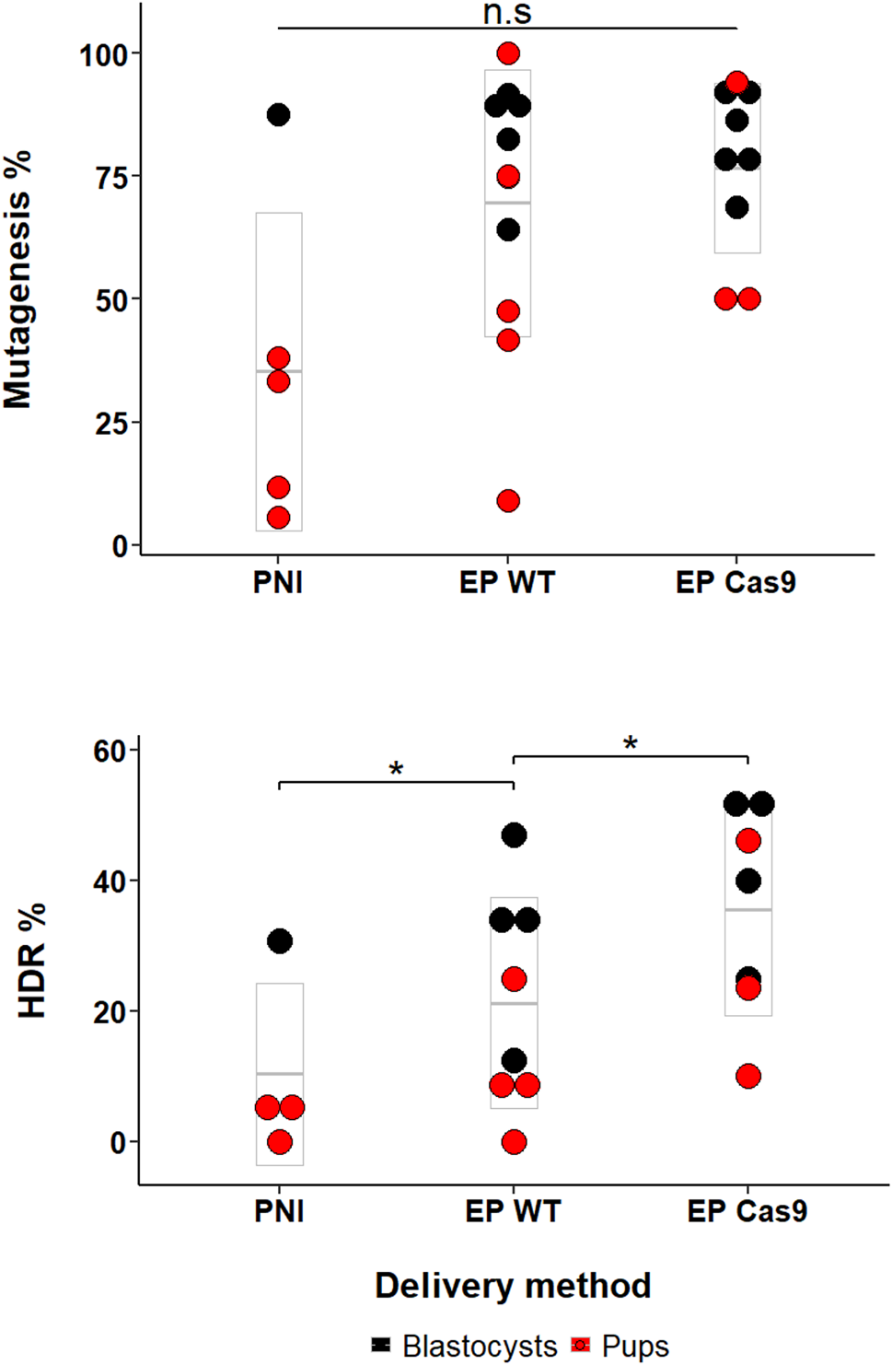
Overall mutagenesis rates for the three delivery methods across all genes tested. Dot plot showing the range of efficiencies for total mutagenesis (upper panel) and knock-in allele production by homology directed repair (lower panel), combining data from both live pup generation and in vitro cultured blastocysts, across all targeted gene loci. CRISPR/Cas9 reagents have been delivered either by pronuclear microinjection of C57BL/6J embryos (PNI), by electroporation of C57BL/6J zygotes (EP WT) or by electroporation of zygotes derived from Cas9-expressing donor females (EP Cas9). The mean is shown by the horizontal line, and the box indicates plus / minus the standard deviation. *p < 0.05.

However, for the production of knock-in alleles, we were able to conclude a statistically significant increase in efficiency of 58% was occurring when using maternally supplied Cas9 zygotes (β = 0.4583 *p* value = 0.0325) as opposed to exogenous Cas9 supply to C57BL/6J zygotes. The distribution of mutagenesis efficiencies (both overall mutagenesis rate and the average rate of knock-in allele production) across these experiments and the 3 methods is summarized in Fig. 6.

### Electroporation yields further production improvements

**Figure 7:**
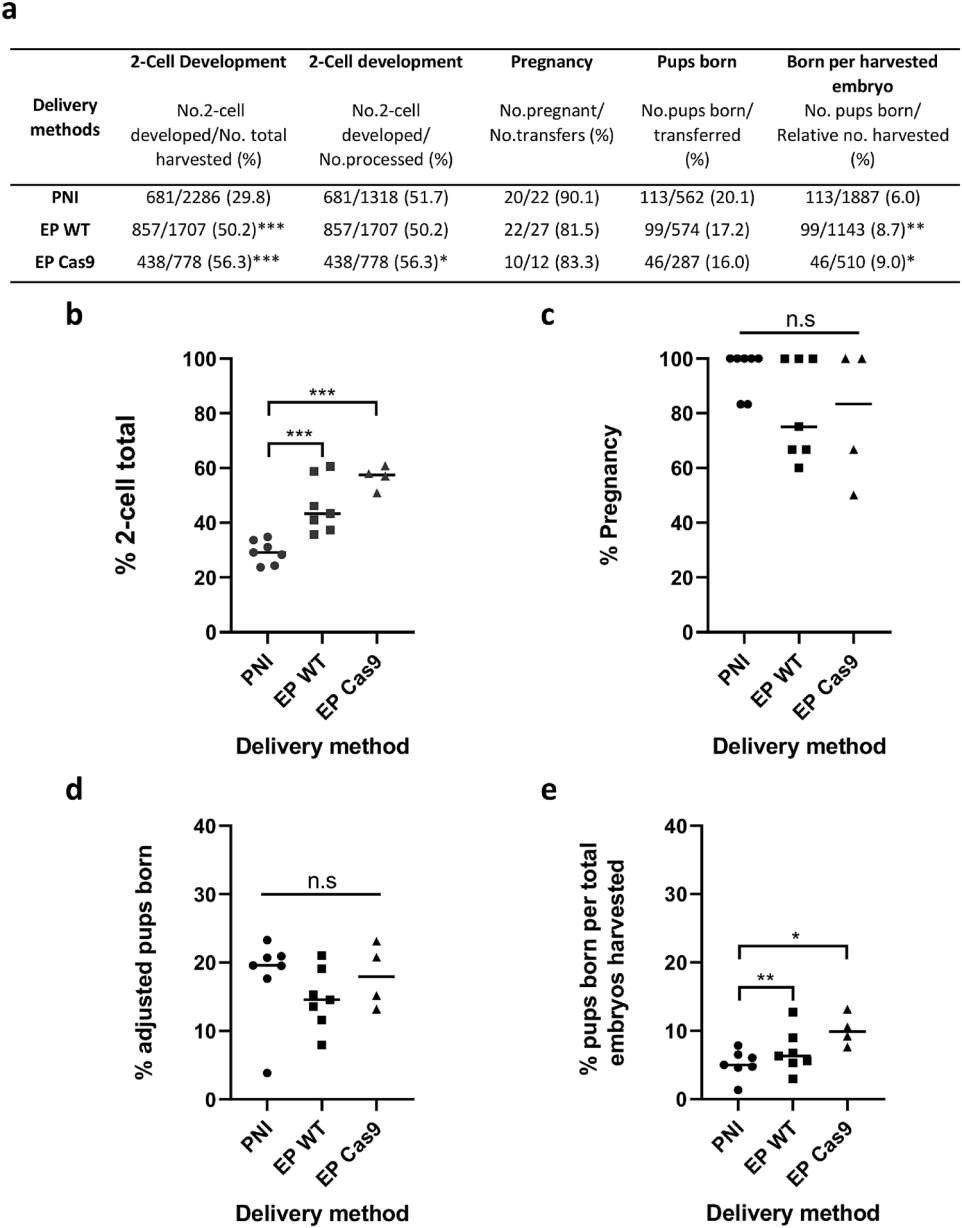
Production efficiencies following pronuclear microinjection versus electroporation. (**a**) Table summarizing embryo and pup survival and production rates following the introduction of CRISPR/Cas9 reagents either by pronuclear microinjection of C57BL/6J zygotes (PNI) or electroporation of wild-type C57BL/6J zygotes (EP WT) or zygotes derived from Cas9-expressing females (EP Cas9). Comparison bar charts showing the rate of 2-cell development relative to the total number of zygotes harvested (**b**), the rate of embryo transfer recipient females that became pregnant (**c**), the rate of pups born, adjusted for non-pregnant transfers (**d**) and the rate of pups born relative to the total number of embryos harvested (**e**). *p < 0.05, **p < 0.01,***p < 0.001 versus microinjection by a two sided Fisher's exact test.

It has previously been suggested that electroporation can be considered a milder intervention when compared with microinjection and subsequently improvements in embryo survival^10^, two-cell progression^5^ and birth rate^10^ have been reported following electroporation. In agreement, we observed that electroporation led to a very significant improvement in embryo survival when compared with pronuclear microinjection (Fig. 7a, Supplementary Table 7, *p* < 0.001). In particular, when assessing the number of two-cell embryos resulting from overnight culture of electroporated or microinjected embryos as a percentage of the initial total number of zygotes harvested, electroporation led to a substantial improvement in production efficiency (Fig. 7b, Supplementary Table 7, *p* < 0.001). Moreover, maternally supplied Cas9 embryos electroporated with sgRNA/ssODN alone showed a further improvement in the rate of two-cell progression when compared to wild-type embryos electroporated with RNP/ssODN (Fig. 7b, Supplementary Table 7; *p* = 0.0049).

Pregnancy rate and birth rate were calculated, the latter being adjusted to exclude non-pregnant females (Supplementary Table 7) and no significant differences in the pregnancy rate (Fig. 7c) or birth rate (Fig. 7d) were observed between our delivery methods (Fig. 7a, Supplementary Table 7). To investigate whether electroporation could have an impact on overall pup production efficiency and thus the number of embryos required to generate a genetically modified animal, we calculated the rate of live pups born as a percentage of total embryos harvested (Supplementary Table 7). The rate of pups born per zygote harvested was higher when electroporation was used compared to microinjection, and the improvement was significant whether we used exogenous Cas9 or maternally supplied Cas9 (*p* = 0.0064 and *p* = 0.02, respectively; Fig. 7e, Supplementary Table 7).

## Discussion

We present a side-by-side analysis comparing the effect that the CRISPR/Cas9 delivery mode (electroporation vs. microinjection) into C57BL/6J zygotes has upon the efficiency of mutagenesis, both for the production of indel and knock-in alleles. Addressing multiple genetic loci, we observe that delivery of CRISPR/Cas9 components by electroporation compares favourably with delivery by conventional pronuclear microinjection, and report that electroporation increases the generation efficiency of knock-in alleles. A further increase in production efficiency for this class of alleles was found when combining electroporation with maternally supplied Cas9.

The increase in efficiency may be due to the electroporation delivery method being more consistent than microinjection. The success of the microinjection procedure is heavily influenced by the technical skill of the technician performing the manipulations and presumably there is a large variation in the treatment that each individual embryo receives, both in terms of physical damage but also in terms of the quantity of injected material. This variation could lead to a significant percentage of microinjected embryos receiving either too much (and therefore a potentially toxic dose) or too little of the reagents for efficient mutagenesis. In contrast, electroporation would be expected to dose each embryo equally. The variability of microinjection when using C57BL/6 strain embryos is also likely to exacerbate differences. The pronuclei of C57BL/6 embryos are difficult to microinject, compared with other strains and hybrids, and accordingly embryos of C57BL/6 strains show the lowest birth rate and percentages of transgenesis per injected zygote^19^. Subsequently, it is perhaps to be expected that electroporation which produces a milder and more consistent delivery of CRISPR/Cas9 reagents would have a significant impact on this genetic background.

The efficiency improvements we observed were only seen for the generation of knock-in alleles which suggests that the repair template could be responsible for these toxic effects. Another potential explanation for increased knock-in efficiency could relate to delivery of CRISPR reagents and ssODN being to both pronuclei when using electroporation whilst when using pronuclear injection typically only one of the two pronuclei is injected.

Many of the comparison have been performed with the same batch of embryos, although due to production variations sometimes this was not possible. Although we cannot rule out the influence of individual batch effects where comparison experiments were not performed on the same day on the same batch of embryos, the large number of experiments reported will hopefully compensate for any batch effects. To reduce experimental variation, electroporation and pronuclear injection were also performed at the same time of day in relation to the hCG hormone injection. However, we cannot rule out an effect caused by the procedural timings of the two delivery methods, as electroporation of all embryos can occur within a few minutes, whereas microinjection of an equivalent number of embryos requires several hours. The impact of the cell cycle on repair efficiencies is well established^20^ and subsequently timing differences may contribute to the efficiency improvements reported in this study.

With respect to embryo processing, electroporation was found to be far less damaging to the embryos than pronuclear microinjection as indicated by a significantly improved embryo recovery rate after manipulation. Furthermore, electroporation is suitable for all fertilized embryos, whereas pronuclear microinjection can only be performed on fertilized embryos where pronuclei have clearly formed. Subsequently, a far greater proportion of the total harvested embryos resulted in viable 2-cell embryos for transfer into recipient females when using electroporation as the delivery method. Following embryo transfer, however, no significant differences were observed in pregnancy rates and pups birth rates with either of the two delivery methods, suggesting no significant impact of the delivery method on the subsequent embryonic development, in agreement with previous studies^13^. Overall, pup production is more efficient with electroporated embryos than microinjected embryos, and thus electroporation has the potential to reduce the animal cost of model production by reducing the absolute number of embryos required for the generation of a genetically modified mouse model.

Cytoplasmic injection has been reported as an alternative delivery method for CRISPR/Cas9 reagents to the fertilized oocyte^21^ and has also been associated with increased survival^22^, as damage to the nuclear membranes is avoided. Furthermore, cytoplasmic microinjection, similar to electroporation, does not require the formation of discernible pronuclei, increasing the proportion of embryos that can be manipulated. How electroporation and cytoplasmic delivery would compare in terms of mutagenesis efficiency and embryo survival would be an interesting question to be addressed in a separate side-by-side comparison. Electroporation, however, is a lot less laborious than microinjection, and requires less specialized equipment and expertise. With electroporation, hundreds of embryos can be processed in a few minutes, whereas any kind of microinjection, including the simplified cytoplasmic delivery, requires at least an hour of technically demanding processing^21^.

The use of Cas9-expressing female mice obviates the need to introduce exogenous Cas9 protein to the zygote and all that is required for delivery are the relatively small sgRNA and ssODN repair template. Previous studies have demonstrated that nucleic acid molecules of this size are freely able to penetrate 1-cell zygotes through the pores opened by the electroporation process^23^. Extrapolating from biophysical experiments examining pore size in electroporated cells^24^, the penetration of the zygote by these small nucleic acids would be expected to be more efficient than for the 160 kDa Cas9 protein at the field strength used in these experiments. The relative inefficiency of delivering the bulky Cas9 protein might thus explain the improved efficiency when using maternal Cas9 supply. However, another explanation could be simply that a higher local concentration of Cas9 is achieved by maternal deposition. In either case, why this would only influence the efficiency of knock-in allele generation remains unclear.

Persistent Cas9 expression has been linked to an increased risk of off-target mutagenesis^25,26^ and consequently, concerns have been raised about the risks of non-specific effects when using mice which ubiquitously express Cas9. We believe this not to be a concern for the following reasons—firstly, in the absence of an activating gRNA, the enzyme is inert and Cas9-expressing mice have no detectable phenotype^14,15,27,28^, and no evidence of increased off-target mutagenesis has been reported^14,15^. Secondly, the use of heterozygous female donor mice means that 50% of the offspring no longer carry the Cas9 transgene and thus the Cas9 within these zygotes is only transiently present—maternal protein is quickly degraded and diluted by subsequent embryo cleavage events, restricting the Cas9 activity as a natural pulse precisely when the Cas9 is required. Furthermore, founder mice which haven’t inherited the Cas9 transgene can be preferentially used to establish the required lines, reducing the risk of any as yet unknown negative consequence of Cas9 expression.

More complex manipulations by direct zygote microinjection have been demonstrated using long-single-stranded DNA templates^29^ and the production of both floxed and fluorescent cassette knock-in alleles has been achieved at high efficiency^30^. Whether electroporation is a suitable delivery method for these types of manipulation remains unclear. One recent study showed in principle that floxed models could be generated by electroporation of long single-stranded DNA templates^31^, however the efficiencies achieved were low, perhaps suggesting that electroporation may not be the most appropriate strategy when using large templates for the production of complex alleles.

In conclusion, the increased knock-in efficiency and the improved in vivo survival of electroporated embryos compared to microinjected embryos, suggest that electroporation should be the delivery method of choice for the generation of genetically modified models, in particular on a C57BL/6J background. The improvements seen suggest that electroporation in combination with maternal Cas9 supply could have an important impact on the number of mice required for the production of genetically modified mouse models. The method thus has the potential to contribute to a significant 3Rs impact, reducing the animal cost of the production of mouse models, in particular for those harbouring simple knock-in alleles.

## Methods

### Preparation of RNPs and repair templates

sgRNAs were designed against the genomic targets using the CRISPOR algorithm^32^ (Supplementary Table 2) and either prepared by in vitro transcription with the EnGen kit (NEB cat# E3322S) followed by purification with the RNA Clean & Concentrator kit (Zymo Research cat# R1013) or synthesized by Synthego Corp or IDT Inc. RNP was prepared by complexing the sgRNA with recombinant NLS-Cas9 protein. ssODN homology repair templates (Supplementary Table 3) were synthesized as 139nt sequences with 5′ and 3′ phosphothioate end protection and PAGE purified (Eurogentec).

### Zygote microinjection and electroporation

3 week old C57BL/6J (Charles River) or heterozygous *Gt(ROSA)26Sor*^*tm1(CAG-cas9)Wthg*^ female mice^14^, were superovulated and mated with wild-type C57BL/6J studs. Fertilized oocytes were prepared from plugged females and microinjected into a pronucleus with 20 ng/µl sgRNA and 100 ng/µl recombinant NLS-Cas9 protein. Where required, ssODN templates for homology directed repair were added to the microinjection mix at a final concentration of 43 ng/µl. Alternatively, zygotes in batches of up to 100 were electroporated, in Opti-MEM media (ThermoFisher Scientific cat# 31985062) containing either 130 ng/µl sgRNA (for oocytes derived from transgenic CAG-Cas9 females) or 130 ng/µl sgRNA and 650 ng/ul recombinant NLS-Cas9 protein (for zygotes derived from wild-type females). Where required, ssODN templates for homology directed repair were added to the electroporation mix at a final concentration of 430 ng/µl. Electroporation was performed with the previously described conditions^7^ (2 square-wave pulses of 30 V, 3 ms duration and 100 ms interval) using a GenePulser Xcell electroporator (Biorad) and a 1 mm electrofusion slide (BLS cat# GSS-1000) or, on a few occasions, in 1 mm electroporation cuvettes (Bio-rad). The timing of microinjection and electroporation were performed between 22 and 24 h after the hCG injection. Microinjected or electroporated zygotes were cultured overnight to the two-cell stage and surgically implanted into recipient pseudopregnant CD1 females, or were cultured in vitro in KSOM-AA medium until blastocyst stage.

### Genotyping and quantification of mutagenesis within embryos

In vitro cultured blastocysts or ear biopsies from the resulting offspring were lysed using standard conditions and genomic DNA purified. The target gene was amplified using the primers listed in Supplementary Table 2, denatured, reannealed and analysed on a 15% PAGE gel to detect the formation of heteroduplexes indicative of indel mutations^33^. For knock-in alleles, restriction endonuclease digestion was used to determine successful homology directed repair followed by Sanger sequencing to confirm the presence of the mutation. Where ambiguous mixed traces were obtained, the PCR product was cloned into the pCR2.1-TOPO TA cloning kit (ThermoFisher Scientific cat# K4550-01) and multiple plasmids sequenced to confirm the presence of the required knock-in mutation.

### Animal work

All animal studies received ethical approval from the Clinical Medicine AWERB (Animal Welfare and Ethical Review Body) at the University of Oxford and were performed in accordance with UK Home Office Animals (Scientific Procedures) Act 1986 under project license PPL PAA2AAE49. Mice were housed in individually ventilated cages and received food and water ad libitum. All surgery was performed under isoflurane inhalation anaesthesia using appropriate analgesia.

### Statistical analysis

The efficiencies of the experimental methods were tested using a mixed-effect logistic regression model. Each result was assigned a set of indicator variables to label which experimental method was used and which gene was attempted to be mutated. The experimental method (microinjection, electroporation using exogenous Cas9, electroporation using maternally supplied Cas9) and the observation method (genotyping of in vitro cultured blastocysts or pups) were treated as fixed-effects and the genes as random-effects. The model was then fit using the function glmer (family = binomial) from the R package lme4^34^. Gene by gene, embryo survival, pregnancy and birth rate comparisons were performed using a two-sided Fisher's exact test. Comparison of the overall mutagenesis and knock-in efficiency for the different delivery methods (genotyping of pups or blastocysts analysed independently) was performed using the Chi-square test. Data sets were analysed and presented using Graphpad Prism software v8.4.

## Supplementary information

Supplementary Information 1

## Data Availability

All data generated or analysed during this study are included in this published article (and its Supplementary Information files). The Cas9-expressing mouse strain used in this study will be deposited in the European Mouse Mutant Archive.
