# Supplementary material for: Electroporation and genetic supply of Cas9 increase the generation efficiency of CRISPR/Cas9 knock-in alleles in C57BL/6J mouse zygotes
Source: Sci Rep. 2020 Oct 21;10:17912. doi: 10.1038/s41598-020-74960-7 (PMC7578782; doi:10.1038/s41598-020-74960-7)
Supplement: Supplementary file 1 — Supplementary Information 1 [file 41598_2020_74960_MOESM1_ESM.pdf]

Electroporation and genetic supply of Cas9 increase the generation efficiency of CRISPR/Cas9 knock-in alleles in C57BL/6J mouse zygotes

Samy Alghadban[1,3], Amine Bouchareb[1,3], Robert Hinch[2], Polinka Hernandez-Pliego[1], Daniel Biggs[1], Chris Preece[1] and Benjamin Davies[1]*

[1]Wellcome Centre for Human Genetics, University of Oxford, Roosevelt Drive, Oxford, OX3 7BN UK

[2]Big Data Institute, Li Ka Shing Centre for Health Information and Discovery, University of Oxford, Oxford, OX3 7LF UK

[3] contributed equally to the work

* to whom correspondence should be addressed

Cas9 conc. (ng/µl)	No. Exp	HDR	Cas9 source	No. harvested	No. used	No. survived	No. 2-cell	2-cell %	No. blast[h]	Blast/ survived %	blast/ 2-cell %	HDR	Mut	Total analysed
650	2	HDR	pCas9	254	109	95	68	71.6%	24	25.26%	35.29%	10	19	24
400	2	HDR	pCas9	237	104	89	56	62.9%	22	24.72%	39.29%	9	16	22
200	2	HDR	pCas9	239	136	117	70	59.8%	21	17.95%	30.00%	9	11	21
100	2	HDR	pCas9	240	150	137	72	52.5%	32	23.36%	44.44%	22	28	32

Supplementary Table 1: Optimization of the Cas9 concentration used for pronuclear microinjection. Table shows the mutation rate and the production statistics following pronuclear microinjection of RNP/ssODN into C57BL/6J zygotes, using Cas9 concentrations varying from 100 ng/µl up to the concentration used in the electroporation experiments (650 ng/µl). [h]: Harvested at the blastocyst stage for analysis of genotypes; pCas9 (Cas9 protein).

Target Locus	Rep. colour	Protospacer 5'-3'	Genomic coordinates	Forward Genotyping Primer	Reverse Genotyping Primer
**Gene A**		GATGGTGATGGTGCGCCAGG	Chr19: 6,276,725-6,300,096 (+)	ACTCCCCTGCTTGGATAGTCT	CCTTGTCCACATCTGCCAAC
**Gene B**		GTGCCTCGTAGTCATCACAC	Chr19: 27,322,588-27,337,179 (+)	CTCACAAGCCTCCTGGATGG	TCCATAGAAGCGCATGTCCC
**Gene C**		GCATGCCTTCAGGCTGACAT	Chr18: 1,438,983-1,483,170 (+)	CTGGCAAAGTCCTGCAGTTG	ACATTGGTCCCTATGGTGGT
**Gene D**		GGGGTAGGAAGTGCACCAAC	Chr19: 51,440,203-51,441,340 (+)	GCTCAGCCAAAGATGAAGAAGGTC	TTCCTCCCAGCTCCAGTTGTTCCA
**Gene E**		CCCAGGCCCAACAGCCATGG	Chr10: 131,984,081-131,989,316 (+)	CAGGGAAGGCTGCGAAGG	CGAATGGTTGGGTCACCAGA
**Gene F**		CATGGTGCAGGACATTTCAC	Chr17: 15,543,079-15,564,354 (-)	AGGAATCTTCCTTCC TTGCTGTCG	ATTTCCCTGTATCTTCTTCAGGACT
**Gene G**		CTGCAGTCGGCCGAAGCTGA	Chr9: 111,228,228-111,271,791 (-)	CTCTCAGTCGGGGAACTCGG	AATAGGAAGAGCGGACCGTG
**Gene H**		ACGGCTGCAAGGGGTTCTTC	Chr2: 167,587,166-167,653,303 (+)	AACCTCAATTCATCCAACAGCC	TTATAGTACAGGCTTGCCATGGA
**Gene I**		GGGCCTGTGTGATGACTACG	Chr19: 27,322,588-27,337,179 (+)	CTCACAAGCCTCCTGGATGG	TCCATAGAAGCGCATGTCCC
**Gene J**		AGTGCTGACTGTAGCATGTG	Chr 6: 115,978,186-115,978,784 (-)	CATGCGCACTCCACTTAGGA	GCAGGTGGAGTGTACCTCAC

Supplementary Table 2: Targets for CRISPR/Cas9 mutagenesis together with their genomic coordinates and the primer sequences used for genotyping

Target Locus	Rep. colour	Template length (nt)	Template sequence	Restriction enzyme
Gene A		139	CCAAGCTGCTGGATACAGTGGATGATATGCTGGCCAACGATATAGCTCGGCTGATGGTGATGGTGTGGCA<br>GGAAGAGTCCCTGATGCCCTCACAGGCTGTGAAGGGTGGTGCTTTTGATGGCACCATGAATGGGCCCTT	EarI
Gene B		139	AAACGCGCCTGGGTCGCCTGGCCACCTCCACCACTCGCAGAGGCCAGCTGGGtCTGTGTGATGACTACTAG<br>GCACAGACAGACGAGTACTTCTTTGACCGTGACCCAGCGGTCTTCCAGCTCATCTACAACTTCTACAC	BfaI
Gene C		139	CAAAGGTTTAGTCCGTTATCATTATGGTAGGAAGCATGCCTTCAGGCTGAAGCTTATAACTTCGTATAATGT<br>ATGCTATACGAAGTTATCATTGGAGCAGTAGCTGAAAACTACATCCTGATCCTTAGGTGGACAGACA	HindIII
Gene F		139	TAGTTACCTCTTCATCCATAGGACCATAGGACCCTGGACCTCTCCAGTGAAATGGCATACCCATACGACGTC<br>CCAGACTACGCTATGTCCTGCACCATGAACACCAACAAGCTGGAAGAAAATAGTCCTGAAGAAGATA	AatII
Gene G		139	AACGCCATATTGGCGCCCGAGTTCCCAGTCAGCCAATCACTCGAGCTCCTCACGCTCAAGTTCTTCCTTTAG<br>CTTCGGCCGACTGCAGTCCTGCGCCGTCTTCCCAGCGGCAAGTGCTGATTGGGCAGCATGAATGCCA	HpyAV
Gene H		139	CGACCGGGCCACCGGCAAACACTACGGAGCCTCGAGCTGTGACGGCTGCAAGGGGTTCTTCTGGAGatctG<br>TGAGaAAGAACCACATGTACTCCTGCAGGTGAGGAGCCAGCTCGGGCCTTAACTTTCTCATCTGGGAT	BglII

Supplementary Table 3: ssODN repair templates and the diagnostic restriction enzyme using for detection of a successful knock-in mutation

Gene ID	No. exp	HDR	Delivery mode	Cas9 source	No. harvested	No. used	No. survived	No. 2-cell	2-cell %	No. Transfers*	Pups born % No. pups/ No.transferred(%)	HDR	Mut	Total analysed
Gene A	2	HDR	EP[a]	pCas9	255	255	253	157	62.1%	3	6/76(7.9)	0	6	6
Gene A	3	HDR	EP[a]	embryos	266	266	262	154	58.8%	5	17/129(13.2)	4	16	17
Gene B	1	HDR	PNI	pCas9	120	83	72	34	47.2%	1	6/34 (17.6)	0	2	6
Gene B	2	HDR	EP[a]	pCas9	216	216	203	127	62.6%	5	24/118 (20.3)	2	10	24
Gene B	2	HDR	EP[a]	embryos	158	158	154	96	62.3%	2	10/66 (15.2)	1	1[c]	10
Gene C	2	HDR	PNI	pCas9	271	112	104	79	76.0%	3	17/82 (20.7)	1	2	17
Gene C	1	HDR	EP[a]	pCas9	180	180	180	83	46.1%	2	4/66 (6.1)	1	3	4
Gene D	2	HDR	PNI	pCas9	374	208	145	91	62.8%	3	18/92 (19.6)	-	1	18
Gene D	2	HDR	EP[b]	pCas9	230	230	219	86	39.3%	3	11/81 (13.6)	-	1	11
Gene E	1	HDR	PNI	pCas9	254	161	143	120	83.9%	4	21/120 (17.5)	-	8	21
Gene E	1	HDR	EP[a]	pCas9	124	124	124	87	70.2%	3	21/76 (27.6)	-	10	21
Gene E	1	HDR	EP[a]	embryos	64	64	63	40	63.5%	1	6/26 (23.1)	-	3	6
Gene F	1	HDR	PNI	pCas9	273	224	185	136	73.5%	3	22/90 (24.4)	1		22
Gene F	1	HDR	EP[a]	pCas9	50	50	50	31	62.0%	1	11/34 (32.4)	1	-	11
Gene F	1	HDR	EP[a]	embryos	132	132	130	62	47.7%	2	13/66 (19.7)	6	7	13

Supplementary Table 4: Genotypes of pups at several targeted loci with different delivery methods. Table summarizing production and genotyping data of pups generated by delivery of CRISPR/Cas9 reagents either by pronuclear microinjection (PNI) or electroporation (EP) into C57BL/6J zygotes using protein Cas9 (pCas9)/ssODN, or electroporation of zygotes derived from Cas9-expressing donor females (embryos) with sgRNA/ssODN. *Number of transfers is equal to the number of pregnant females. [a] Electroporation using a 1mm slide; [b] Electroporation in a 1mm cuvette; [c] Only 2 of the 10 pups have been assessed for mutagenesis.

Gene ID	No. exp	HDR	Delivery mode	Cas9 source	No. harvested	No. used	No. survived	No. 2-cell	2-cell %	No. blast[h]	Blast/ survived %	Blast/ 2-cell %	HDR	Mut	Total analysed
Gene A	4	HDR	PNI	pCas9	597	355	295	143	48.5%	65	22.0%	45.5%	20	28	65[a]
Gene A	4	HDR	EP	pCas9	302	302	280	175	62.5%	119	42.5%	68.0%	56	89	119
Gene A	2	HDR	EP	embryos	134	134	134	79	59.0%	53	39.6%	67.1%	27	49	53
Gene B	1	No	EP	embryos	29	29	29	18	62.1%	9	31.0%	50.0%	-	7	9
Gene B	3	HDR	EP	embryos	179	179	175	131	74.9%	44	25.1%	33.6%	-	33	43
Gene G	1	No	EP	pCas9	100	100	97	40	41.2%	31	32.0%	77.5%	-	30	31
Gene G	1	HDR	EP	pCas9	83	83	79	50	63.3%	26	32.9%	52.0%	9	17	26
Gene G	1	No	EP	embryos	30	30	30	15	50.0%	11	36.7%	73.3%	-	8	11
Gene G	1	HDR	EP	embryos	104	104	102	76	74.5%	40	39.2%	52.6%	21	27	40
Gene H	1	HDR	EP	pCas9	70	70	67	47	70.1%	39	58.2%	83.0%	13	25	39
Gene H	1	HDR	EP	embryos	104	104	102	73	71.6%	40	39.2%	54.8%	16	32	40
Gene I	1	No	EP	pCas9	75	75	65	28	43.1%	21	32.3%	75.0%	-	29	32
Gene I	1	HDR	EP	embryos	105	105	99	62	62.6%	37	37.4%	59.7%	-	32	37
Gene J	1	HDR	EP	pCas9	158	158	150	70	46.7%	24	16.0%	34.3%	3	22	24
Gene J	1	HDR	EP	Embryos	45	45	44	20	45.5%	12	27.3%	60.0%	3	11	12

Supplementary Table 5: Genotypes of blastocysts at several targeted loci with different delivery methods. Table summarizing production and genotyping data of blastocysts generated by delivery of CRISPR/Cas9 reagents either by pronuclear microinjection (PNI) or electroporation (EP) into C57BL/6J zygotes using protein Cas9 (pCas9)/ssODN, or electroporation of zygotes derived from Cas9-expressing donor females (embryos) with sgRNA/ssODN.

	Mutagenesis				HDR			
	PNI vs EP WT		EP WT vs EP Cas9		PNI vs EP WT		EP WT vs EP Cas9	
	Pups	Blastocysts	Pups	Blastocysts	Pups	Blastocysts	Pups	Blastocysts
p values	0.0222	0.426	0.015*	0.6543	0.3023	0.165	0.0163	0.0461

Supplementary Table 6: Impact of the delivery methods on mutagenesis and HDR efficiency, analysed within experimental group (genotyping of pups or blastocysts). Summary of statistical analysis, using Chi-square test, of the different delivery methods [pronuclear microinjection (PNI) or electroporation into C57BL/6J zygotes (EP WT) using protein Cas9 (pCas9)/ssODN, or electroporation of zygotes derived from Cas9-expressing donor females (EP Cas9) with sgRNA/ssODN] to establish whether the mode of delivery has any effect on the mutagenesis or knock-in (HDR) rate. This test has been used to perform comparisons on pups and blastocysts separately. In comparing the impact of the delivery methods, it was striking that the above p-values are highly variable within an experiment type (genotyping of blastocysts or genotyping of pups), indeed the variable efficiencies across different gene loci weakens this analysis method (*in particular). Subsequently for a thorough analysis of this highly structured data, a designed model was developed to take into consideration the experiment type and the gene locus as variables. This model has been used to draw the main conclusions of the study and is presented in **Fig. 6** (please refer to the main text).

Gene ID	No. exp	Delivery mode	Cas9 source	No. harvested	No. used	No. survived	No. 2-cell	% 2-cell (total)	% 2-cell (survived)	No. transferred	No. transfers	No. Pregnant (%)	No. Pups born	% born (adjusted for n.p.)	% born per total harvested embryos
Gene B	1	PNI	pCas9	120	83	72	34	28.3%	47.2%	34	1	1 (100%)	6	17.65%	5.00%
Gene C	2	PNI	pCas9	271	112	104	79	29.2%	76.0%	82	3	3 (100%)	17	20.73%	6.04%
Gene D	2	PNI	pCas9	374	208	145	91	24.3%	62.8%	92	3	3 (100%)	18	19.57%	4.76%
Gene E	2	PNI	pCas9	505	285	248	170	33.7%	68.5%	170	6	5 (83%)	33	23.29%	7.84%
Gene F	4	PNI	pCas9	731	435	349	227	31.1%	65.0%	172	6	5 (83%)	30	20.93%	6.50%
Gene H	1	PNI	pCas9	173	113	95	41	23.7%	43.2%	41	2	2 (100%)	8	19.51%	4.62%
Gene I	1	PNI	pCas9	112	82	76	39	34.8%	51.3%	26	1	1 (100%)	1	3.85%	1.34%
Gene A	3	EP[a,b]	pCas9	441	441	430	241	54.7%	56.0%	202	7	4 (57%)	15	13.00%	7.10%
Gene B	2	EP[a]	pCas9	216	216	203	127	58.8%	62.6%	118	5	5 (100%)	24	20.34%	11.96%
Gene C	1	EP[a]	pCas9	180	180	180	83	46.1%	46.1%	66	2	2 (100%)	4	6.06%	2.79%
Gene D	2	EP[b]	pCas9	230	230	219	86	37.4%	39.3%	81	3	3 (100%)	11	13.58%	5.08%
Gene E	2	EP[a]	pCas9	327	327	275	198	60.6%	72.0%	119	5	5 (100%)	25	21.01%	12.72%
Gene F	2	EP[a,b]	pCas9	195	195	194	80	41.0%	41.2%	74	3	1 (33%)	11	44.59%	18.30%
Gene H	1	EP[a]	pCas9	118	118	109	42	35.6%	38.5%	42	2	2 (100%)	9	21.43%	7.63%
Gene A	3	EP[a]	embryo	266	266	262	154	57.9%	58.8%	129	5	5 (100%)	17	13.18%	7.63%
Gene B	2	EP[a]	embryo	158	158	154	96	60.8%	62.3%	66	2	2 (100%)	10	15.15%	9.21%
Gene E	2	EP[a]	embryo	128	128	127	73	57.0%	57.5%	52	2	1 (50%)	6	23.08%	13.16%
Gene F	2	EP[a,b]	embryo	226	226	212	115	50.9%	54.2%	94	3	2 (67%)	13	20.74%	10.56%

Supplementary Table 7: Impact of the delivery method on the production of live pups. Summary table for the embryo transfer statistics following delivery of CRISPR/Cas9 reagents either by pronuclear microinjection (PNI) or electroporation (EP) into C57BL/6J zygotes using protein Cas9 (pCas9)/ssODN, or electroporation of zygotes derived from Cas9-expressing donor females (embryos) with sgRNA/ssODN. This table includes extra data from pups that have not been genotyped (not shown in Supplementary Table 4). [a] Electroporation on slide; [b] Electroporation in cuvette; n.p. not pregnant

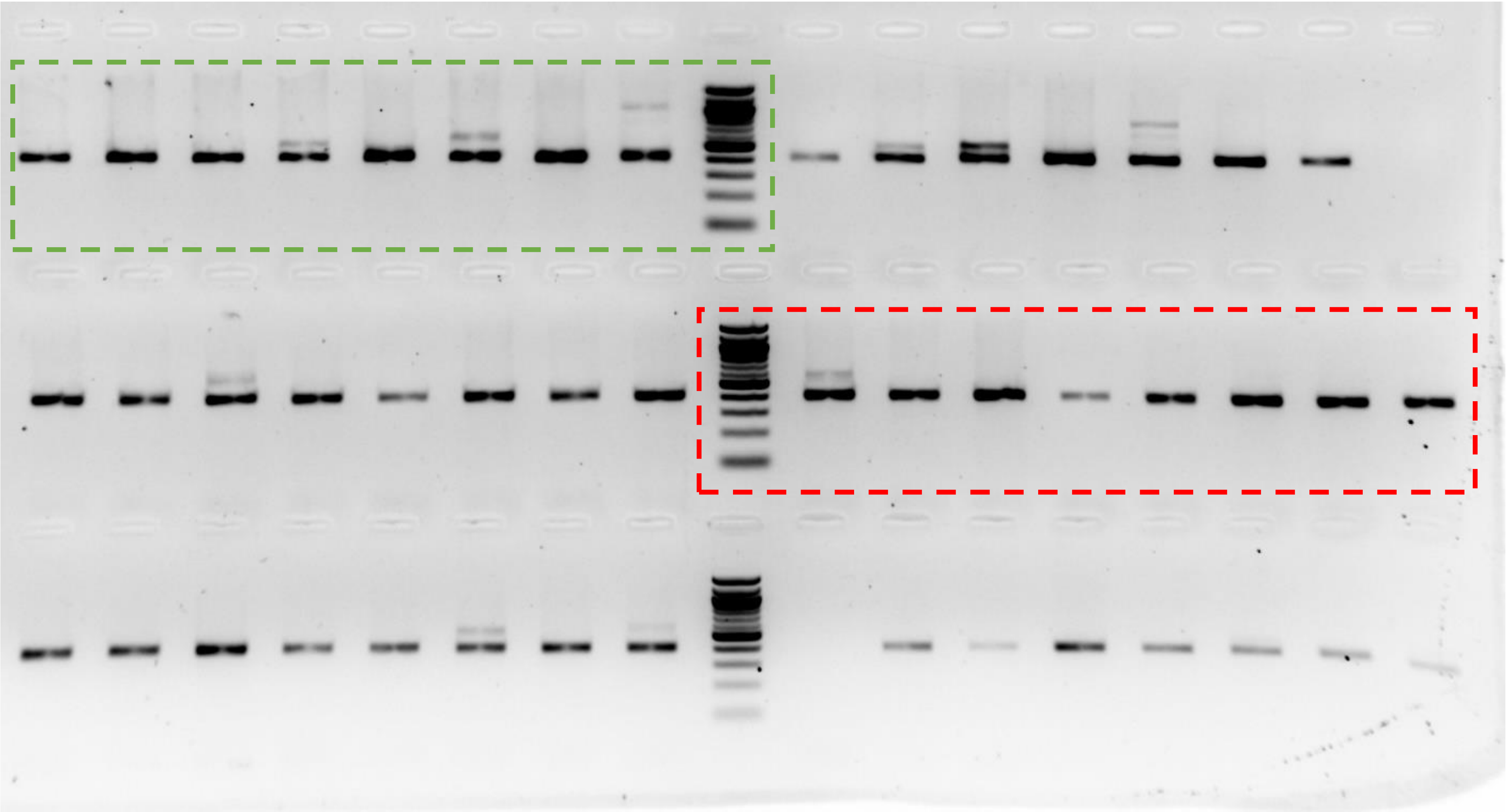

Original gel for Figure 1 (top panel) “PCR gel 2% Agarose”
2 examples are shown – left hand portion is shown above in red outline, right hand portion is shown above in green outline

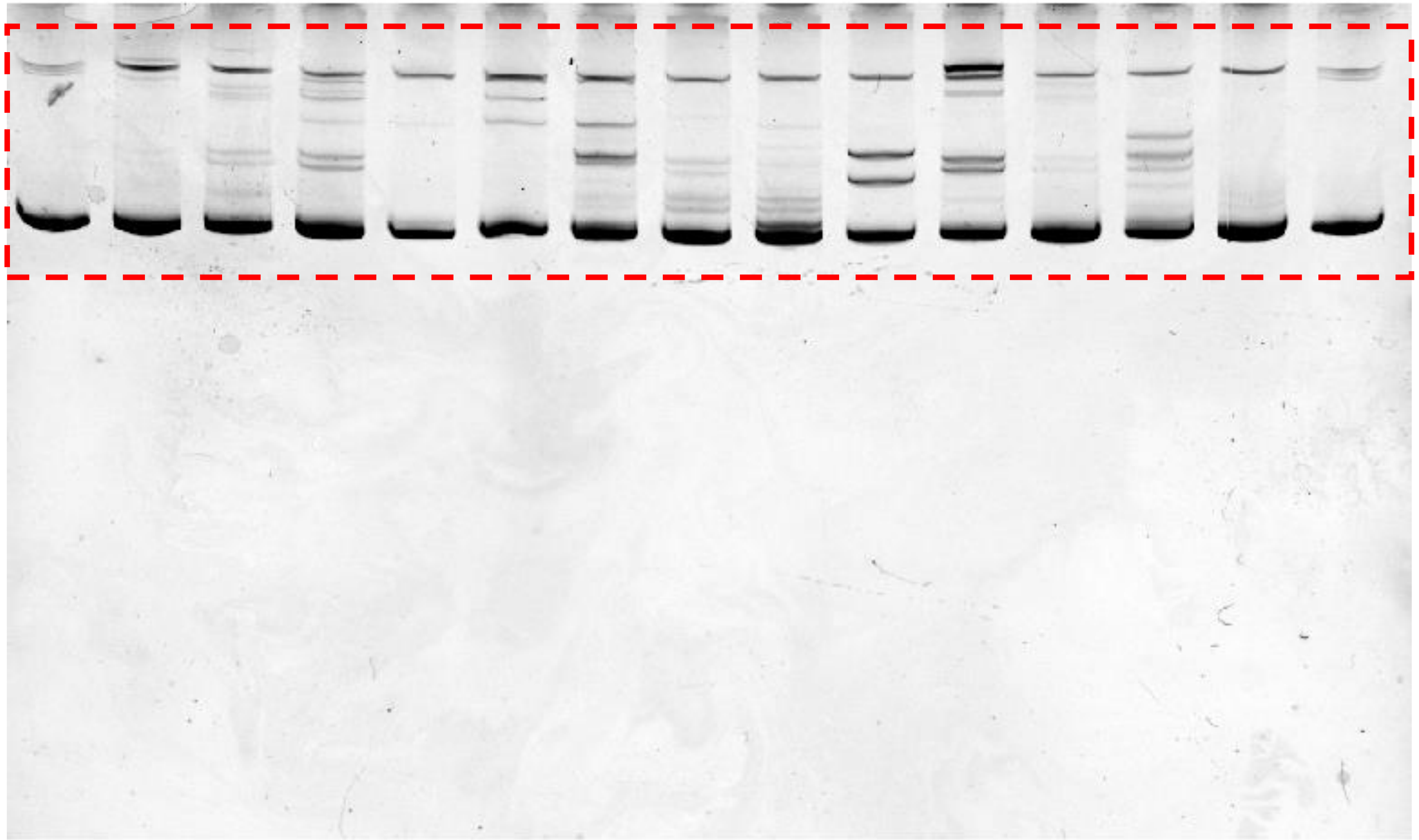

Original gel for Figure 1 (middle panel) “Heterodimers 15% PAGE”

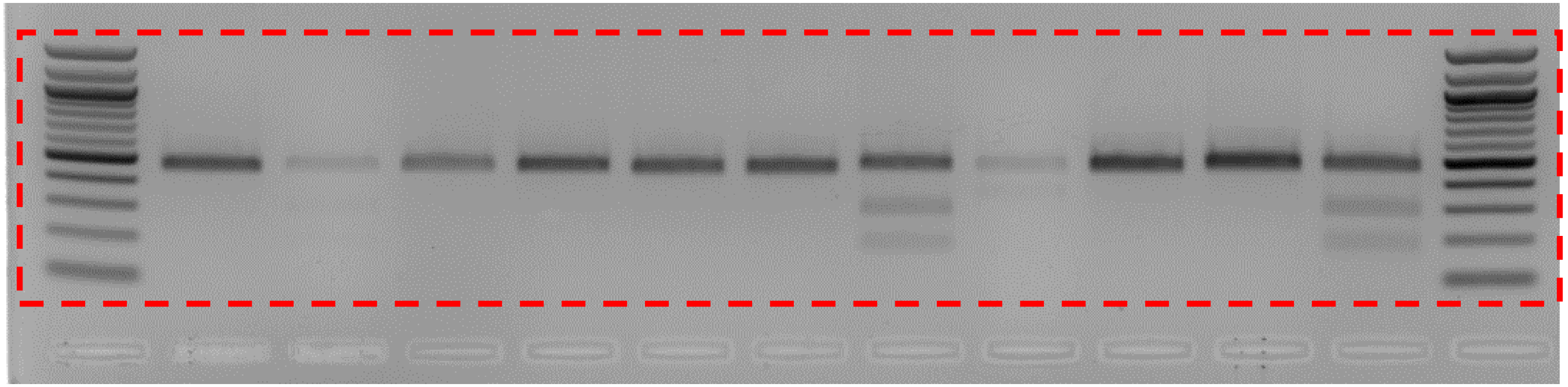

Original gel for Figure 1 (lower panel) “PCR digest gel 2% Agarose”
